# Identification of Novel Biomarkers and Candidate Drug in Ovarian Cancer

**DOI:** 10.3390/jpm11040316

**Published:** 2021-04-19

**Authors:** Chia-Jung Li, Li-Te Lin, Pei-Yi Chu, An-Jen Chiang, Hsiao-Wen Tsai, Yi-Han Chiu, Mei-Shu Huang, Zhi-Hong Wen, Kuan-Hao Tsui

**Affiliations:** 1 Department of Obstetrics and Gynaecology, Kaohsiung Veterans General Hospital, Kaohsiung 813, Taiwan; nigel6761@gmail.com (C.-J.L.); litelin1982@gmail.com (L.-T.L.); ajchiang490111@gmail.com (A.-J.C.); drtsai0627@gmail.com (H.-W.T.); m1221226@gmail.com (M.-S.H.); 2 Institute of Biopharmaceutical Sciences, National Sun Yat-sen University, Kaohsiung 804, Taiwan; 3 Department of Obstetrics and Gynaecology, National Yang-Ming University School of Medicine, Taipei 112, Taiwan; 4 School of Medicine, College of Medicine, Fu Jen Catholic University, New Taipei 242, Taiwan; chu.peiyi@msa.hinet.net; 5 Department of Pathology, Show Chwan Memorial Hospital, Changhua 500, Taiwan; 6 Department of Health Food, Chung Chou University of Science and Technology, Changhua 510, Taiwan; 7 National Institute of Cancer Research, National Health Research Institutes, Tainan 704, Taiwan; 8 Department of Microbiology, Soochow University, Taipei 111, Taiwan; chiuyiham@scu.edu.tw; 9 Department of Marine Biotechnology and Resources, National Sun Yat-sen University, Kaohsiung 804, Taiwan; wzh@mail.nsysu.edu.tw; 10 Department of Obstetrics and Gynecology, Taipei Veterans General Hospital, Taipei 112, Taiwan; 11 Department of Pharmacy and Master Program, College of Pharmacy and Health Care, Tajen University, Pingtung County 907, Taiwan; 12 Department of Medicine, Tri-Service General Hospital, National Defense Medical Center, Taipei 114, Taiwan

**Keywords:** ovarian cancer, bioinformatics, CREB1, drug perturbation

## Abstract

This paper investigates the expression of the CREB1 gene in ovarian cancer (OV) by deeply excavating the gene information in the multiple databases and the mechanism thereof. In short, we found that the expression of the CREB1 gene in ovarian cancer tissue was significantly higher than that of normal ovarian tissue. Kaplan–Meier survival analysis showed that the overall survival was significantly shorter in patients with high expression of the CREB1 gene than those in patients with low expression of the CREB1 gene, and the prognosis of patients with low expression of the CREB1 gene was better. The CREB1 gene may play a role in the occurrence and development of ovarian cancer by regulating the process of protein. Based on differentially expressed genes, 20 small-molecule drugs that potentially target CREB1 with abnormal expression in OV were obtained from the CMap database. Among these compounds, we found that naloxone has the greatest therapeutic value for OV. The high expression of the CREB1 gene may be an indicator of poor prognosis in ovarian cancer patients. Targeting CREB1 may be a potential tool for the diagnosis and treatment of OV.

## 1. Introduction

Ovarian cancer is a common malignant tumor in gynecology, with the highest mortality rate and the second highest incidence rate of gynecologic malignancies, and the 5-year survival rate is only 25–30% [1]. Current studies have found that epigenetic modifications play an important role in the occurrence and development of ovarian cancer [2]. Therefore, a comprehensive study on the pathogenesis of ovarian cancer and the establishment of effective prevention and treatment programs are the urgent issues that we should address now. Nowadays, systematic analysis based on gene microarray technology using bioinformatics methods to study tumor-related genes and their regulatory mechanisms is one of the main research tools in functional genomics.

CREB1 is a nuclear protein in eukaryotic cells and a nuclear factor that regulates transcription. It is composed of 341 amino acid residues and has a molecular weight of 43 KDa. It belongs to the CREB/ATF subgroup of the leucine zipper family of transcription factors [3]. CREB is activated by phosphorylation, forming homodimers or heterodimers, and it is regulated by cofactors to recognize and bind the cAMP response element (CRE) in the target gene promoter, promoting the transcriptional expression of the gene and participating in tumor proliferation, differentiation, and metastasis [4]. Extracellular signals interact with receptors on the cell membrane to phosphorylate and activate CREB1 via signaling pathways such as PKA, PKC, PKB, and ERK [5]. It further regulates the cell cycle, promotes cell proliferation, inhibits apoptosis, etc. Several studies have shown that target genes regulated by CREB1 play an important role in cell proliferation, differentiation, survival, and cell cycle regulation. Its overexpression contributes to cell survival and proliferation and has an important role in the development of several tumors [6,7,8,9].

In this study, we first performed bioinformatics analysis to investigate the prognostic and therapeutic impact of CREB1 on OV. We identified human ovarian cancer tissue microarray and different stages of ovarian cancer cells for analysis of CREB1 protein and mRNA levels. Finally, pharmacogenomics was used to predict potential drugs. Since naloxone has been approved for clinical treatment of lung cancer [10], our results may support the use of CREB1 gene status as an ovarian cancer biomarker and precision treatment of OV patients with naloxone.

## 2. Materials and Methods

### 2.1. Cells and Cell Culture

Ovarian cancer cell lines OC-117-VGH cells (BCRC#60601, Hsinchu, Taiwan), OC-117-VGH cells (BCRC#60602), OCPC-2-VGH (BCRC#60603), OC-3-VGH (BCRC#60599), TOV-21G (BCRC#60407), and NIH-OVCAR-3 (BCRC#60551) were used and cultured in DMEM/F12 supplemented with 1.5 g/L sodium bicarbonate and 10% fetal bovine serum (Themo Fisher Scientific, Waltham, MA, USA) in a humidified atmosphere of 95% air and 5% CO_2_ at 37 °C.

### 2.2. RNA Extraction and Real-Time PCR

The total RNA was extracted with the EasyPrep Total RNA Kit (BIOTOOLS Co., Ltd., Taipei, Taiwan). A total of 1 μg of RNA was reverse-transcribed with a ToolScript MMLV RT kit (BIOTOOLS Co., Ltd.) for cDNA synthesis. Real-time PCR was carried out using a StepOnePlusTM system (Applied Biosystems, Foster City, CA, USA) with TOOLS 2X SYBR qPCR Mix (BIOTOOLS Co., Ltd.). The expression levels of all the genes in cells were normalized to the internal control RNU6-1 gene. All the samples with a coefficient of variation for Ct value > 1% were retested.

### 2.3. Tissue microarrays (TMA) and Immunohistochemistry (IHC) Analysis

Tissue array slides (CJ2) containing human ovarian cancer, metastatic, and normal tissues were purchased from SuperBioChips Laboratories (Seoul, Republic of Korea). For immunohistochemistry (IHC), assays and scoring methods were performed as described [11]. The slides were treated with anti-CREB1 antibody (1:100, A11063, ABclonal, Boston, MA, USA). All glass slides were digitized with an Motic Easyscan Digital Slide Scanner (Motic Hong Kong Limited, Hong Kong, China) at ×40 (0.26 μm/pixel) with high precision (High precision autofocus). Motic Easyscan whole-slide images were viewed with DSAssistant and EasyScanner software at Li-Tzung Pathology Laboratory (Kaohsiung, Taiwan).

### 2.4. Multi-Omics Analysis

TumorMap is an integrated genomics portal for visual and exploratory analysis that biologists and bioinformaticians can use to query a rich set of cancer genomics data. The intuitive and interactive layout helps to identify cancer subtypes based on common molecular activities in a set of tumor samples [12].

The gene mutations and co-expression of CREB1 were computed and analyzed using CBio-Cancer Genomics Portal (cBioPortal) databases. Searching the term “CREB1” enabled the acquisition of the full mutation distribution across all tumor and non-tumor tissues. We analyzed the expression of CREB1 in 9736 tumors and 8587 normal tissues using this tool [13].

Gene Expression Profiling Interactive Analysis (GEPIA) is an interactive network database that can be linked and analyzed with other databases (TCGA and GTEx). Using GEPIA, we analyzed 9736 tumors and 8587 normal tissues [13].

Metascape contains fully integrated data from multiple databases such as GO, KEGG, UniProt, and DrugBank. With metascape, it is possible to perform a complete pathway enrichment and biological process annotation, gene-related protein network analysis, and drug analysis [14].

We used Reactome to compare a pathway to its homolog in another species, and the protein–compound interactions from external databases were used to confirm our findings [15,16,17]. The data contained 51,745 PPIs among 10,177 human proteins. After filtering the PPI data for proteins encoded by genes having transcriptome data in TCGA datasets, a network was reconstructed with 34,604 PPIs among 8322 proteins.

The CMap database collects drug-induced gene expression profiles from human cancer cell lines, which can be used to compare the similarity and dissimilarity between the inputted DEGs and drug-induced gene expression [18].

### 2.5. Statistical Analyses

All data were presented as mean ± standard deviation (SD) or case number (%). The correlation between the clinicopathological parameters and the four gene expressions was analyzed using the chi-square or Fisher exact tests for categorical variables, and paired-sample t-test for continuous variables, using the GraphPad Prism 8.0 (GraphPad Software, San Diego, CA, USA). The Spearman rank correlation test was used to analyze the correlation results of expression of the four biomolecules. In this study, the endpoints were overall survival (OS) and disease-free survival (DFS). The results of univariable analysis of the variables and survival data were performed using the Kaplan-Meier method with the log-rank test. The relationship between the variables and survival data was analyzed via Cox’s proportional hazards regression model. Statistical significance was defined as a *p*-value < 0.05.

## 3. Results

### 3.1. TumorMap and Integrated Cluster Identify Significant Features Distinguishing OV among PanCancer-33 Tumors

**Figure 1:**
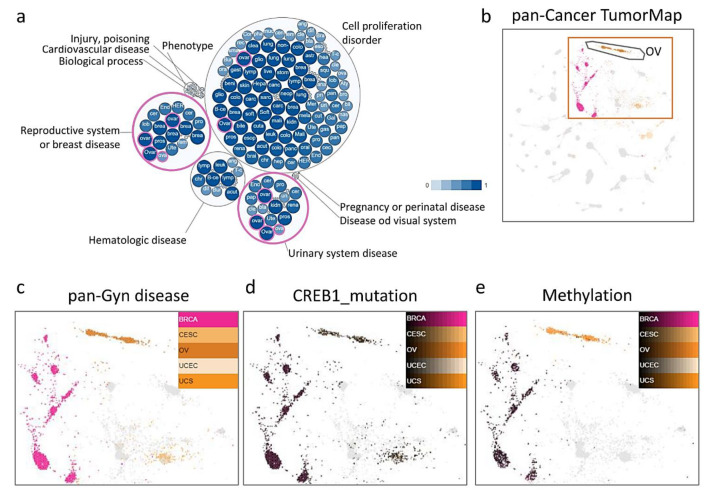
TumorMap and integrated cluster of gynecologic cancers from PanCancer-33 Analysis. (**a**) The distribution of CREB1 in various cancers and physiological functions. (**b**) TumorMap analysis visualizing close mapping of BRCA, CESC, OV, UCEC, and UCS among 28 PanCancer-33 islands. (**c**) Higher resolution view of TumorMap islands and distribution of Gyn cancers from five sites. (**d**) CREB1 mutation status showing the majority of mutation BRCA and OV map around a distinct island. (**e**) Methylation of Gyn cancers. Each spot in the map represents a sample. The colors of the sample spots represent attributes as described for each panel. BRCA: Breast invasive carcinoma, CESC: cervical squamous cell carcinoma and endocervical adenocarcinoma, OV: ovarian serous cystadenocarcinoma, UCEC: uterine corpus endometrial carcinoma, UCS: uterine carcinosarcoma.

First, we explored whether CREB1 is involved in multiple types of cancer and physiological functions. We investigated the sample subgroups revealed by the integration of the multi-omics platform. We categorized the inverse significance of the similarity of the data to create an integrated graph with equal contributions from seven different data platforms. Each group represents a different type of physiological function. Several known connections between tumor types can be seen on the result graph. We found that CREB1 is widely expressed in reproductive system or breast disease and urinary system disease (Figure 1a). To identify a molecular signature-based classification, we conducted an integrated tumor map and cluster identify analysis of 9,759 tumor samples from PanCancer-33 cancers (Figure 1b) for which Gyn disease (Figure 1c), mutation (Figure 1d), and methylation (Figure 1e), and a smaller set of protein expression profiles were available. The integrated map separates OV patients into distinct cytogenetic subgroups, which are characterized by differential cancer types. The OV-like tumors are further characterized by mutations and methylation in CREB1. Therefore, we judged the maps to be biologically relevant.

### 3.2. CREB1 Gene Mutation Predicts A Poorer Disease-Free Survival in OV Patients

**Figure 2:**
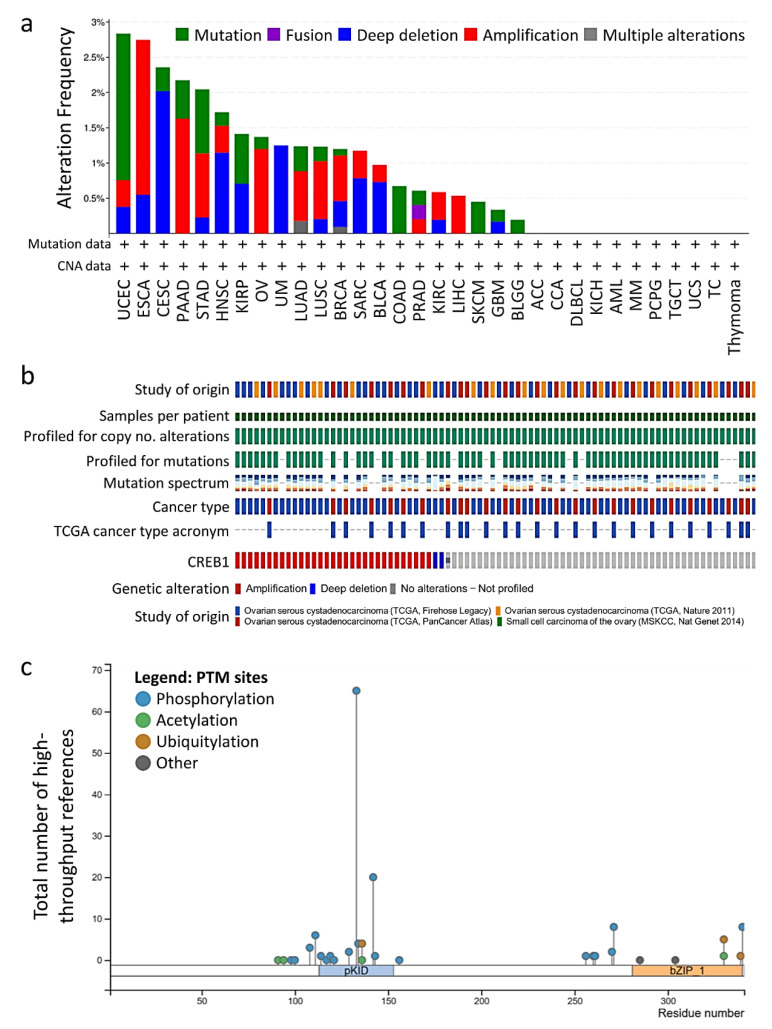
Copy number amplification of CREB1 gene in OV. (**a**) CREB1 gene was analyzed for gene alterations (mutation status and copy number variation) in various cancer types using “TCGA, PanCancer Atlas” data in the cBioPortal cancer genomics database; (**b**) Oncoprint table of significant signature genes. The Oncoprint table summarizes genomic alterations in all queried genes across samples. Red bars indicate gene amplifications, blue bars are homozygous deletions, and green squares are nonsynonymous mutations. (**c**) A cartoon diagram for the gene and protein structures of CREB1. This figure was adapted from the image obtained from the cBioPortal website.

CREB1 expression is frequently found in OV. Indeed, we mined “TCGA, PanCancer Atlas” data via the cBioPortal website for the genetic alterations of CREB1 gene. Among the 32 tumor types we used as dataset, the expression levels of these hub genes varied from 0.17% to 2.08%, and the mutation frequency of each hub gene was shown in Figure 1a. This pan-cancer analysis also indicated that CREB1 gene alterations occurred most frequently in OV, compared with other cancer types (Figure 2a). The alterations for the CREB1 gene was calculated to be between 0% and 2.1% in the examined ALL samples. Genetic mutations of CREB1 were 2.1% (Figure 2b). From the diagram of CREB1 gene and the encoded protein, mutations occurred more frequently in the kinase inducible domain (KID) that is responsible for heteromerization and transactivation (Figure 2c).

### 3.3. Distribution and Expression of CREB1 in Cancer Tissues of Patients with OV

**Figure 3:**
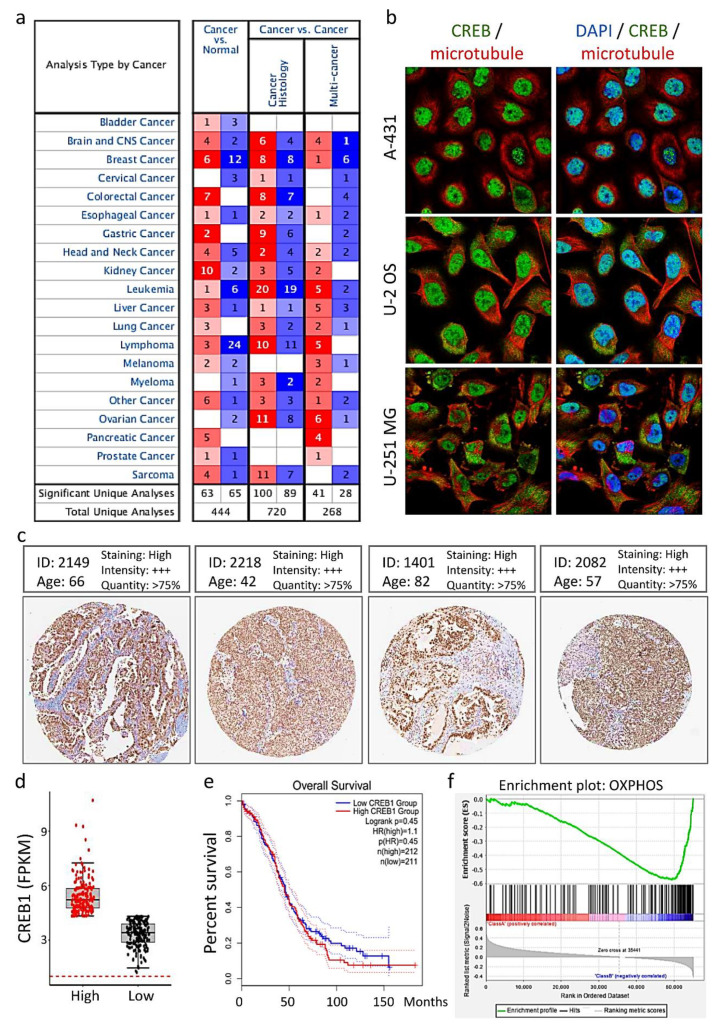
Diagnostic and prognostic value of CREB1 in OV. (**a**) The graphic was generated from the ONCOMINE database, indicating the number of datasets with statistically significant (*p* < 0.01) mRNA overexpression (red) or downregulation (blue) of CREB1. (**b**) The localization of CREB1 protein in human cells. Blue: nucleus; Green: CREB1; Red: microtubules. (**c**) Comparison of immunohistochemistry images of CREB1 in OV tissues with four different patients based on the Human Protein Atlas (+++: strong staining). (**d**) Plots chart showing higher CREB1 expression in OV patients. Data were obtained from TCGA. (**e**) Kaplan–Meier curves was performed to determine differences in OV patients. (**f**) The association between CREB1 gene mutations and OV gene signature. Gene set enrichment analysis (GSEA) was performed to enrich the OV gene signature in the following data sets.

To analyze the expression pattern of CREB1 in various cancers, we accessed the TCGA and GEPIA databases. We carried out the comparison of the transcriptional levels of CREB1 in cancers with those in the normal specimens through the use of ONCOMINE databases (Figure 3a). The significant upregulation of the mRNA expression levels of CREB1 was carried out in OV patients. We further acquired the experimental evidence about the sub-localization of CREB1. Meanwhile, sub-localization of CREB1 in human cell line A-431 and U-251 MG demonstrated that CREB1 protein existed at the nucleus of A-431 and U-251 MG cells41 (Figure 3b). In addition, immunohistochemistry of pathological sections from the Human Protein Atlas Database (HPAD) showed that the protein expression of CREB1 was substantially increased in OV tissues of different ages (Figure 3c). Next, we determined the transcriptional expression of the target genes differentially expressed between OV and normal tissues in TCGA. mRNA levels of CREB1 were found to be significantly increased in OV, indicating that these proteins may have potential carcinogenic effects (Figure 3d). Subsequently, OV patients with high levels of CREB1mRNA expression had low overall survival (Figure 3e). GSEA analysis of RNA-seq data was performed to further explore the involved biological pathways and cofactors of CREB1 in OV. High CREB1 expression was defined as TPM in the 1st quartile, and low FBXW4 expression was defined as TPM in the 4th quartile. The results showed that in the patients with high CREB1 expression, the gene sets were significantly enriched in OXPHOS (normalized enrichment score (NES) = 1.862, *p* = 0.002) (Figure 3f).

### 3.4. Tissue Microarray Analysis of CREB1 Expression

**Figure 4:**
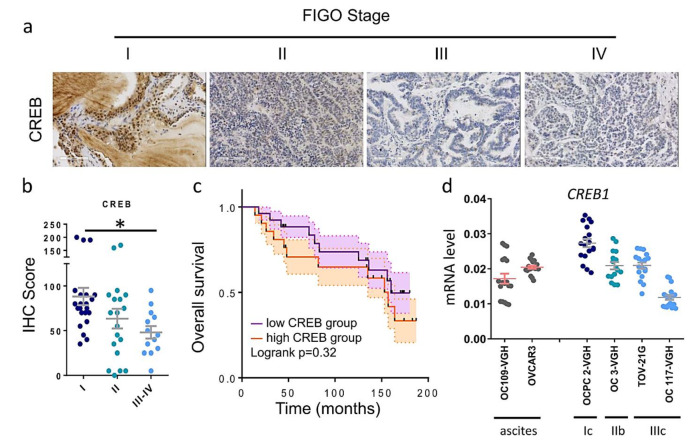
Immunoreactivity of CREB in OV. (**a**) The representative photomicrographs of CREB expression for weak (+), and strong (+++) staining in OV tissues (*n* = 59). (**b**) The IHC score of CREB expression in OV tissue. (**c**) Kaplan–Meier curves were performed to determine differences in OV patients. (**d**) RT-PCR was used to detect the expression levels of different ovarian cancer cells, and U6 small nuclear 1 (RNU6-1) was used as an internal control (*n* = 18). * *p* < 0.05.

To further confirm the accuracy of the multi-omics analysis, we evaluated CREB1 detected using immunohistochemistry in tumor tissues by using 60 OV commercial tissue microarrays. The results of CREB1 expression in OV tissues in IHC staining are shown in Figure 4a. The expression of CREB1 was significantly higher in early stages than in late stages. At higher IHC scores, CREB1 expression was significantly higher in patients with stage I than in patients with advanced stages (*p* < 0.05; Figure 4b). Similar to the above-mentioned TCGA data, the overall survival rate of OV patients with high CREB1 mRNA expression levels was lower than that of OV patients with low CREB1 mRNA expression levels (Figure 4c). Next, we analyzed the endogenous levels of CREB1 in ovarian cancer cell lines, and the results showed that the mRNA expression levels of CREB1 were higher in early-stage cells than in late-stage cells in different ovarian cancer cell lines (Figure 4d).

### 3.5. Prediction of Protein–Protein Interaction of CREB1 Mutations and Copy Number Alterations

**Figure 5:**
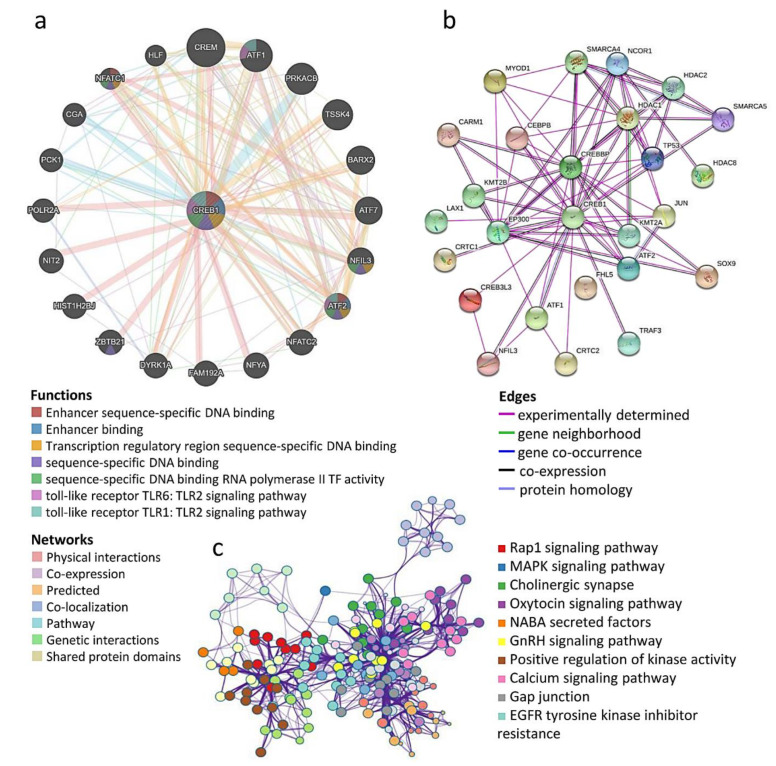
Network data improves the target prediction (**a**) and (**b**) predicted PPI essential for the functions of CREB1 generated from Pathway Commons and String website. (**c**) Metascape functional enrichment analysis and OV clinical relevance of CREB1-regulated DEGs. One term per cluster, colored by *p* values. Log10 (p) is the *p* value in log base 10.

Next, we conducted Metascape Pathway and process enrichment analysis integrating the gene ontology sources, including GO Biological Process, KEGG pathway, Reactome Gene Sets, and Canonical Pathways. The predicted protein partners of CREB1 were ATF1, ATF2, PRKACB, TSSK4, BARX2, ATF7, NFIL3, NFATC2, NFYA, FAM192A, DYRK1A, ZBTB21, HIST1H2BJ, NIT2, POLR2A, PCK1, CGA, NFATC1, HLF, SOX9, FHL5, JUN, and LAX1. Thus, these predicted interacting protein partners of CREB1 might be involved in the regulation of CREB1-mediated cancer progression and prognosis (Figure 5a). Protein–protein interaction clustering algorithm identified neighborhoods within the networks where the CREB1-regulated genes were densely connected, such as ATF1, ATF2, NFATC1, NFIL3, TP53, JUN, SOX9, etc. (Figure 5b). Top 20 clusters were defined with their representative enriched terms (Figure 5c), including MAPK signaling pathways, calcium signal, and the EGFR pathway. Furthermore, network enrichment captured the interactions between the 20 clusters, as visualized using Cytoscape. These results revealed the novel and essential biological functions of CREB1 in multiple molecular pathways. As shown in Figure 3d, in addition to OV, the high alteration frequency of the CREB1 gene was also found in pancreatic adenocarcinoma (PAAD), esophageal carcinoma (ESCA), lung adenocarcinoma (LUAD), lung squamous cell carcinoma (LUSC), and breast invasive carcinoma (BRCA). The cancer genomics (mutations, copy number variations, and mRNA levels) and patients’ survival data in these cancer types were analyzed for the role of CREB1.

### 3.6. Naloxone Treatment Mimics the Gene Expression Profile of CREB1

**Figure 6:**
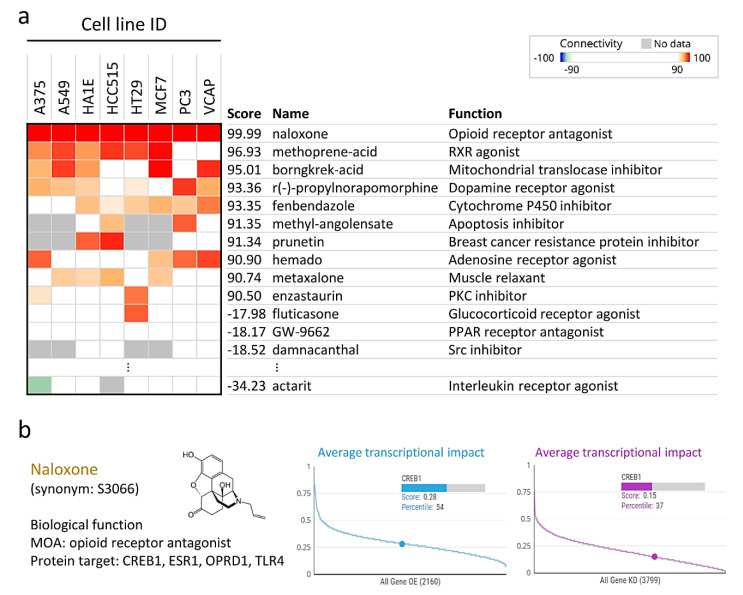
CMap analysis and drug sensitivity profiling in ovarian cancer cells. (**a**) The CREB1 gene signature was queried using the CMap database to predict potential drugs to reverse this signature. (**b**) The correlation between CREB1 gene overexpression and knockdown in OV cell lines (The Cancer Therapeutics Response Portal CTRP-OV data from the CTRP database).

To investigate whether naloxone could target CREB1 functional activity, we employed the CMap analysis. The CMap database allows users to query a gene signature and explore the connections between the queried gene signature and drug-driven gene expression [19]. We prepared the differentially expressed genes (DEGs) from CREB1-overexpressing different cancer cells and queried the CMap database. Figure 4a shows the top 10 perturbagens that mimicked the CREB1-driven gene signature, including naloxone with the median connectivity score of 99.9. In contrast, the median connectivity score of fluticasone is −17.98. Therefore, naloxone treatment may mimic the effect of CREB1 overexpression. We further searched the OE and KD gene libraries of 2160 and 3799 oncogenes from the pharmacogenomic database and searched for potential drugs for the treatment of ovarian cancer. As shown in Figure 6b, we found a positive correlation between naloxone and CREB1 OE score of 0.28 (*p* < 0.05); and CREB1 KD score of 0.15. This indicates that CREB1 gene overexpression is positively correlated with naloxone sensitivity. The average transcriptional impact showed a positive correlation between CREB1 mRNA expression and naloxone drug activity. The above result implied that naloxone may reverse the CREB1-associated cancer hallmarks.

## 4. Discussion

Ovarian cancer is one of the most prevalent malignant tumors in women. With a high degree of malignancy and a short survival period, ovarian cancer is often detected at an advanced stage, and the current treatment is still based on surgery and chemotherapy [20]. Current studies have shown that the abnormal expression of several genes may be closely associated with the survival of ovarian cancer patients through the screening of gene expression profiles, and that these genes are involved in the development and progression of ovarian cancer by promoting or suppressing apoptosis, generating or reversing chemotherapy resistance [21,22]. In this study, we used bioinformatics analysis, ovarian cancer tissue microarray, and multiple types of ovarian cancer cell lines to screen out mRNAs that may be related to the prognosis of ovarian cancer patients and provide a basis for future clinical practice and scientific research.

Previous studies have demonstrated that CREB1 expression levels have an important role in ovarian granulosa cell survival and that follicle-stimulating hormone and luteinizing hormone regulate ovarian function, at least in part, through the cAMP intracellular signaling pathway [23]. Somers et al. found that the mutation of Ser l33 to Ala l33 in the CREB1 sequence, which results in an inability to be activated by phosphorylation, significantly inhibits murine ovarian granulosa cell survival [24]. This also suggests that CREB1 is an important protein that promotes ovarian survival and plays an important role in the development of ovarian cancer [19,25]. Tumor development is not only about the activation of oncogenes and inactivation of oncogenes but also about abnormal expression of apoptosis-related genes and overexpression of growth factors and uncontrolled cell cycle. Most tissue cells undergo malignant transformation with shortened cell cycle and accelerated proliferation.

In addition to reducing metastasis and cell proliferation in bladder cancer cells, it also reduces gastric cancer, esophageal cancer, and glioma by knocking down CREB1 gene levels in tumor cells [26,27,28,29]. It is also possible to inhibit CREB1 activity through pharmacological strategies. Previous studies have shown that treatment of cancer cells with 666-15, a CREB inhibitor [30], has potent anticancer activity both in vitro and in vivo. Imperatorin also directly targets CREB1 to inhibit TGFβ2-ERK signaling and inhibit esophageal cancer metastasis [29].

Although CREB1 has been extensively studied in various tumors [31], CREB1 is aberrantly expressed in a variety of human cancers, including solid tumors [9,31,32,33] and hematologic malignancies [34,35]. In breast cancer studies, CREB1 was found to be highly expressed in metastatic breast cancer cells compared to non-metastatic cells and promoted breast cancer metastasis and subsequent bone destruction [9]. CREB1 was also highly expressed in glioma tissues through the induction of oncogenic microRNA-23a expression and increased the growth survival of glioma cells and colorectal cancer [32,36]. However, there is still evidence that CREB1 inhibits the proliferative effects of the stress-induced acetylcholinesterase variant AChE-R in glioblastoma [33], suggesting a controversial or tissue-specific role for CREB1 in human cancers. In our results, more specifically, there is a significant difference between stage I and stage III/IV, which is directly related to the limited number of patient examinations, in addition to the heterogeneity of both patient and tumor stage that may affect the results. In the future, more ovarian cancer specimens should be collected for confirmation.

In this study, we observed that the expression of CREB1 is dysregulated in pan-cancer, especially in OV. Our study has provided a more detailed picture of the relationship between CREB1 expression and characteristics, prognosis, protein–protein interaction, and hub genes and pathway crosstalk in OV. Our results indicate that CREB1 is highly expressed in OV through tissue microarrays and human ovarian multiple cancer cell lines. Kaplan–Meier analysis indicates that CREB1 may be a potential prognostic factor for OV. Similar to our results, Xu and colleagues reported that CREB1 can be used as a predictor of the prognosis of esophageal cancer [29]. Another study showed that CREB1 plays a vital role in the tumor progression of upper liver cancer [37]. In addition, CREB1 has also been reported to be directly related to the ability of colon, breast, and gastric cancer to metastasize [28,38,39]. These findings showed that CREB1 could serve as a novel biomarker for OV cancer diagnosis and prognosis prediction.

This study has some limitations. First, only in silico and in vitro experimental analyses were performed. Further investigations using animal models specific for OV were required. Second, only 59 cases of OV patients were available for TMA analyses. Third, in this study, a gene expression signature-based approach was used in different cell lines, which should further validate the protein levels as reflecting the results of patient IHC. The differences of genetic mutations, epigenetics, proteomics, and metabolomics should also be considered in future investigations. Finally, although this study was verified by multi-omics, it still requires a large number of clinical specimens and further confirmation through multiple centers.

## 5. Conclusions

In conclusion, our study shows for the first time that CREB1 is ectopically expressed in OV and is a potential new biomarker for OV survival, providing valuable information to guide research on targeted treatment strategies. Our results warrant further investigation into the mechanisms by which CREB1 promotes tumor progression and metastasis in OV.

## Data Availability

Not applicable.
